# Noncontrast T1 values do not correlate to clinically relevant variables in patients with hypertrophic cardiomyopathy

**DOI:** 10.1186/1532-429X-15-S1-P106

**Published:** 2013-01-30

**Authors:** Ethan Rowin, Martin Maron

**Affiliations:** 1 Hypertrophic Cardiomyopathy Center, Tufts Medical Center, Boston, MA, USA

## Background

Hypertrophic cardiomyopathy (HCM) is a genetic heart disease characterized by diffuse histopathologic abnormalities involving the entire LV myocardium, including an expanded extracellular matrix due to replacement and interstitial fibrosis and myocyte disarray. T1 mapping is a novel CMR imaging sequence which characterizes abnormal myocardial tissue composition in cardiomyopathies such as HCM. Therefore, we sought to examine the relationship between T1 values and a variety of relevant demographic, clinical and imaging variables in a cohort of HCM patients.

## Methods

CMR imaging with late gadolinium enhancement and T1 mapping using a noncontrast Shortened Modified Look-Locker Inversion recovery (ShMOLLI) method was performed in 15 HCM patients (56±16 years; 80% male). After obtaining a left ventricular short-axis stack with complete ventricular coverage, T1 values were calculated for each slice and a mean T1 value for the entire myocardium was obtained. T1 values were then compared to a number of relevant demographic, imaging and clinical variables.

## Results

The mean T1 value of the global LV myocardium was 860 ± 54 ms (range 768-977 ms). There was no relationship between global T1 values and a number of relevant HCM variables including: age (p= 0.43), maximal LV wall thickness (p=0.35), LV mass index (p=0.50), presence or extent of LGE (p= 0.07 and 0.50, respectively), heart failure symptoms (874 ms in NYHA class I vs. 860 ms NYHA class II-IV; p=0.62), or between patients with or without resting LV outflow tract obstruction (853 vs 878 ms; p=0.38). Even when comparing mean T1 values of one mid-LV or basal short-axis slice, no significant relationship was present between these HCM variables (p>0.05 for mid and basal slices).

With respect to clinical markers of increased risk, T1 values were not significantly different among HCM patients with markedly increased LV mass index (>91g/m^2^ in males; >69 g/m^2^ in females) as compared to those with normal LV mass (867 vs 857 ms; p=0.77) or in HCM patients with substantial LGE (>10% of LV myocardium) compared to those without LGE (860 vs 819 ms; p= 0.08).

## Conclusions

In patients with HCM, noncontrast T1 mapping using the ShMOLLI method demonstrated no significant relationship with a variety of clinically significant HCM clinical and imaging variables, including markers of increased risk such as extensive LGE and extreme hypertrophy. Although additional investigations are necessary in larger populations, these results suggest that T1 values may not represent a relevant clinical marker for risk assessment in patients with HCM.

## Funding

n/a

